# Silicalite Nanosheet Laminated Membranes: Effects of Layered Structure on the Performance in Pervaporation Desalination

**DOI:** 10.3390/membranes16010032

**Published:** 2026-01-04

**Authors:** Xinhui Sun, Yukta Sharma, Landysh Iskhakova, Zishu Cao, Junhang Dong

**Affiliations:** 1 Department of Chemical and Environmental Engineering, University of Cincinnati, Cincinnati, OH 45221, USA; sunxh@ucmail.uc.edu (X.S.); sharmayu@mail.uc.edu (Y.S.); iskhaklr@mail.uc.edu (L.I.); 2 Jasper Department of Chemical Engineering, The University of Texas at Tyler, Tyler, TX 75799, USA

**Keywords:** silicalite nanosheet, membrane, pervaporation, brine desalination, lithium recovery

## Abstract

Silicalite nanosheet (SN) laminated membranes are promising for pervaporation (PV) desalination of concentrated brines for water purification and critical material concentration and recovery. However, scaling up the SN-based membranes is limited by inefficient synthesis of monodispersed open-pore SN single crystals (SNS). Here, we report a scalable approach to fabricate multilayered silicalite nanosheet plate (SNP) laminated membranes on porous alumina and PVDF substrates and demonstrate their excellent PV desalination performance for simulated brines containing lithium and high total dissolved salts (TDS). At 73 ± 3 °C, the SNP laminated membrane on alumina support achieved a remarkable water flux ( $J_{w}$ ) of nearly 20 L/m^2^·h, significantly outperforming the alumina-supported SNS laminated membrane ( $J_{w}$ = 9.56 L/m^2^·h), while both provided near-complete salt rejection ( $r_{i}$ ~99.9%) when operating with vacuum pressure on the permeate side. The PVDF-supported SNS and SNP laminated membranes exhibited excellent $J_{w}$ (14.0 L/m^2^·h) and near-complete $r_{i}$ (>99.9%), surpassing the alumina-support SNP laminated membranes when operating by air sweep on the permeate side. However, the $r_{i}$ of the PVDF-supported membranes was found to decline when operating with vacuum pressure on the permeate side that was apparently caused by minimal liquid permeation through the inter-SNP spaces driven by the transmembrane pressure. With scalable SNP production, SNP-A membranes show potential for PV desalination of high-TDS solutions, especially in harsh environments unsuitable for polymer membranes.

## 1. Introduction

Crystalline microporous zeolite membranes are promising for reverse osmosis (RO), pervaporation (PV), or membrane distillation (MD) desalination of various brines containing high total dissolved salts (TDS) to recover freshwater and critical materials [1,2,3,4,5]. Defect-free zeolite membranes, because of their size-exclusion ion rejection mechanism at the uniform sub-nanometer zeolitic pore entrances, are capable of achieving near perfect salt rejection for feed brines with TDS up to saturation [6,7,8]. Compared to polymeric membranes, which have been recently reported to achieve near-complete salt rejection with extraordinary water flux in PV desalination of high-TDS solutions [9], zeolite membranes offer unique advantages of extraordinary resistance to hydrothermal environments, acids, bases, dissolved organics, and radioactive elements associated with various sources of wastewater and brine containing critical materials [10,11]. However, the traditional polycrystalline zeolite membranes formed by in situ hydrothermal crystallization or seeded secondary growth methods rely on in-plane crystal growth to close up the intercrystalline gaps. This causes thickness increase by simultaneous out-of-plane growth, leading to excessive resistance to molecular transport in zeolitic channels and low water flux (*J_w_*; L/m^2^·h) [12]. Moreover, the fragility of the traditional zeolite polycrystalline thin films requires the use of expensive, low-packing-area, and resistive rigid inorganic supports, thus further hindering practical applications.

The recent success in synthesis of zeolite nanosheets (ZNs) with very large side-to-thickness aspect ratios ( l/δ > 10^2^) have enabled ultrathin zeolite membranes with preferential zeolitic channel orientations to simultaneously shorten the diffusion length and reduce non-selective intercrystalline spaces, thus improving both the mass transport rate and separation selectivity [6,13,14]. We previously demonstrated the synthesis of high-performance MFI-type ZN-laminated membranes (ZNLMs) on alumina substrates [12] and subsequently developed a method for ZNLM fabrication on low-cost porous polymer substrates [6,13]. MFI zeolites possess a 3D channel system consisting of nearly cylindrical straight channels (0.56 nm × 0.53 nm) along the b-axis and slightly elliptical channels (0.55 nm × 0.51 nm) laying zigzag in the a–c plane. These pore openings are slightly smaller than the kinetic diameters (d_k_) of hydrated metal ions and common anions (d_k_ > 0.6 nm) but are much bigger than the d_k_ of water molecules (∼0.27 nm). Because the ion hydration numbers and hence the hydrated ion kinetic sizes are nearly independent of the TDS and temperature, defect-free MFI zeolite membranes are capable of ion-sieving desalination of concentrated brines [6,15,16]. The membranes demonstrated great PV desalination performance and ion separation selectivity for battery applications. However, for laminating ZN membranes on polymers, the ZNs must be preactivated and mono-dispersed in liquid medium by complex procedures. To avoid irreversible ZN agglomeration during high temperature calcination to open the zeolite pores, the as-synthesized templated nanosheets were first coated on a polymer substrate and activated by ultraviolet (UV) irradiation for several days [17]. The nanosheets were then redispersed in solvent to form a suspension suitable for membrane coating. This complex process causes an extremely low yield of dispersed open-pore ZNs and thus discourages commercial considerations.

A high degree of nanosheet dispersion is essential for forming defect-free, preferentially oriented multilayered thin membranes. In contrast, agglomerates create larger voids and cause misaligned nanosheet deposition. Such defective microstructures diminish membrane separation performance, an undesirable phenomenon also reported in other nanosheet-layered membranes [18]. Recently, we have established a method for synthesis of flower-like pure-silica MFI-type zeolite (silicalite) nanosheet (SN) assemblies consisting of unprecedentedly large-sized multilayered SN petals [19]. The open architecture of the SN flowers allows for template-removal by high-temperature calcination (>450 °C) without causing the spatially separated SN petals to collapse. The activated open-pore SN assemblies were then mechanically disintegrated in liquid phase to directly form suspension of dispersed flat SN plates (SNPs). This well-dispersed SNP suspension was subsequently reformulated for laminating membranes on porous substrates. In this work, we demonstrate the fabrication of SNP laminated membranes on both porous alumina and polyvinylidene difluoride (PVDF) substrates and evaluate their performance for PV desalination and concentration of lithium-containing brines with high TDS.

## 2. Experimental

### 2.1. Chemicals and Materials

The chemicals and materials used in this work included tetraethyl orthosilicate (TEOS, >99%, Sigma-Aldrich, St. Louis, MO, USA), tetrapropylammonium hydroxide (TPAOH, 1 M aqueous, Sigma-Aldrich), hydroxypropyl cellulose (HPC, 99%, Mw. 100,000, Sigma-Aldrich), sulfuric acid (95–98%, Sigma-Aldrich), sodium hydroxide (99.99%, Sigma-Aldrich), sodium chloride (≥99.5%, Fisher Scientific, Waltham, MA, USA), magnesium sulfate (≥99.5%, Sigma-Aldrich), lithium chloride (≥99%, Sigma-Aldrich), potassium chloride (≥99%, Sigma-Aldrich), 1,5-diaminopentane (98%, Alfa Aesar, Ward Hill, MA, USA), iodopropane (99%, Sigma-Aldrich), 2-butanone (≥99.0%, Sigma-Aldrich), potassium carbonate (anhydrous, ≥99%, Sigma-Aldrich), silicic acid (99.9%, 20 μm, Sigma-Aldrich), ethyl acetate (anhydrous, 99.8%, Sigma-Aldrich), ethanol (>99.5%, Sigma-Aldrich), methanol (>99.8%, Sigma-Aldrich), and dimethyl sulfoxide (DMSO, 99.8%, Sigma-Aldrich), porous PVDF film (hydrophilic surface, d_p_ ~0.45 µm and $\varepsilon_{PVDF}$ ~80%, TISCH Scientific, Cleves, OH, USA), PVDF powders (Kynar Flex^®^ 2801-00, Arkema Inc., Radnor, PA, USA), and α-alumina powder (SG16, particle diameter ~0.46 µm, Alcoa Co., Pittsburgh, PA. USA). The porous alumina disc supports were home-made by dry-press and high-temperature sintering processes. The discs are typically 2.5 cm in diameter and 1 mm in thickness, possessing an average pore diameter of ~0.1 µm with a porosity of 30 ± 3% and room-temperature N_2_ permeability of about 3.0 × 10^−8^ mol/m·Pa·s.

### 2.2. Material Characterizations

The morphology of the zeolite nanoparticle seeds, the microstructures of the SN assemblies, the SNPs, and the SNP laminated thin membranes were examined by scanning electron microscopy (SEM) and energy dispersive X-ray spectroscopy (EDS) using an FEI (Hillsboro, OR, USA) Scios DualBeam microscope equipped with Ametek (Berwyn, PA, USA) Octane Super EDAX.

### 2.3. SNS and SNP Synthesis

The SNS were synthesized by secondary growth from silicalite nanoparticle seeds in a silicious solution containing diquaternary bis-1,5(tripropyl ammonium) pentamethylene diiodide (dC_5_) as the structure-directing agent (SDA). The dC_5_ was synthesized in-house via the alkylation of 1,5-diaminopentane with 1-iodopropane following the previously reported procedure [13,15]. The synthesis of the silicalite nanoparticle seeds used the procedures described in the same references, which included two steps of in situ crystallization of a precursor with molar compositions of 10 SiO_2_: 2.4 TPAOH: 0.87 NaOH: 114 H_2_O. This solution was prepared by mixing 0.16 g water, 0.127 g NaOH, 8.93 g of 1.0 M TPAOH aqueous solution, and 2.5 g silicic acid in a Teflon flask. After stirring at room temperature overnight, the in situ crystallization was performed in a Teflon-lined autoclave at 50 °C for 6 days under static conditions. The liquid was then filtered with a GHP syringe filter (pore size 0.45 μm). The clear filtrate solution was used for the second hydrothermal crystallization at 100 °C in the Teflon lined autoclave for 3 days. The resultant silicalite nanoparticles of the second crystallization were recovered by alternating the centrifugation and DI water washing process until pH of the suspension is close to neutral.

The SN synthesis was then conducted by seeded secondary growth using the above obtained silicalite nanoparticle seed in a precursor with molar compositions of 80 TEOS: 3.75 dC_5_: 20 KOH: 9500 H_2_O. This precursor was prepared by mixing the constituting chemicals at room temperature. The mass of silicalite nanoparticle seeds subsequently added to the synthesis mixture was 1/200 of the SiO_2_ mass calculated based on the amount of TEOS in the precursor. After mixing, the seed-added precursor was aged at room temperature for 16 h under an Ar purging flow (50 mL/min) to reduce the ethanol content before hydrothermal reaction. The seeded crystallization was then performed in a Teflon-lined autoclave at 140 °C for 4 days. After reaction, the autoclave was quenched with tap water, and the solid products were recovered by centrifugation. The amorphous silica residual in the products was dissolved by dispersing the solids in a solution of 0.1 M KOH + 1 M KCl (pH ~13) for 1 week. The nanosheets were then recovered by centrifugation and then repeatedly washed with DI water until the suspension approached neutral pH. The as-synthesized nanosheets were sonicated for 2 h in water-filled or ethanol with 4 mm diameter ZrO_2_ milling beads to mechanically dissociate the seed-evolved cores from the encircling 4 nm thick flat sheets. The resulting 4 nm thick flat nanosheet flakes, i.e., single-crystal silicalite nanosheets (SNSs), were subsequently separated from core-area thick debris using centrifugal sedimentation. The preparation of dispersed SNS involves 7 major steps that take about 30 days without activation to open the zeolite pores.

The flower-like silicalite nanosheet assemblies were synthesized by hydrothermal secondary growth of a siliceous precursor containing dC_5_ SDA and seeds of the SNSs following our previously reported procedure [19]. The SNS seeds were dispersed in a synthesis precursor solution at a silica-based precursor-to-seed mass ratio of >500. The precursor solution had a molar composition of 80 TEOS: 3.75 dC_5_: 20 KOH: 12000 H_2_O. The relatively dilute synthesis solution was used to avoid undesirable nucleation and growth of conventional crystals that are favored in high-concentration solutions. The seeded crystallization took place in a Teflon-lined autoclave at 140 °C for 4 days. After reaction, the solid products were separated by centrifugation and then thoroughly washed with 0.1 M KOH + 1 M KCl solution before filtration recovery of the solid and drying. The nanosheet assemblies were dried in an oven and then calcined at 500 °C in air for 6 h to remove the SDA (dC_5_) from the zeolitic pores. The activated SN assemblies remained in a flower-like architecture. The calcined SN flowers were suspended in liquid ethanol placed in a bed of 4 mm diameter ZrO_2_ milling beads, which was subsequently sonicated for 2 h to disintegrate the SN assemblies and produce flat SNPs [6]. The non-flat debris from the joint parts of the SN assemblies were removed by sedimentation and centrifugation to achieve a suspension of flat SNPs. The SNPs were suspended in ethanol before being reformulated into suspensions for coating the SNP laminated membrane on the porous PVDF substrates (SNP-P). For fabricating the SNP laminated membranes on the porous alumina supports (SNP-A), a suspension of flat SNPs was obtained from the SN flowers without calcination for pre-activation because the SNP-A was calcined at high temperature (H.T.) to remove the HPC binders and consolidate the layered SNP film through the water condensation reaction between the contacting SNP surfaces, i.e., $\equiv Si-OH+HO-Si\equiv \;\overset{H.T.}{\to }\;\equiv Si-O-Si\equiv +\;H_{2}O$ . It should be noted that subsequent synthesis of the SN assemblies can use the small SNP debris to seed secondary growth. Thus, the SNP suspension can be synthesized by 3 major steps including the activation by calcination within 7 days.

### 2.4. Membrane Fabrication on Alumina and PVDF Substrates

The SNP laminated membranes were coated on two types of substrates, i.e., a porous alumina disc and porous PVDF film, using the vacuum filtration method. The SNP suspension was prepared by mixing the original SNP–ethanol suspension, additional ethanol, and a 0.1 wt.% HPC aqueous solution. The HPC was used as a binder to prevent the coated SNP layer from cracking or peeling during the drying process before calcination for film consolidation. The final suspension contained 0.02 wt.% SNPs and 0.01 wt.% HPC. The 2.5 cm diameter alumina disc was mounted on a filtration apparatus with the ~4.9 cm^2^ surface exposed for membrane coating. The SNP suspension was loaded to the surface of substrate which was pre-soaked with ethanol. The suspension was loaded at a level of 1.7 g suspension per cm^2^ of coating area. The amount of SNP in this loaded suspension would form a 403 nm thick completely dense single crystalline silicalite film. After filtration coating, the wet membrane was dried in an oven at ~40 °C overnight and then calcined in air at 500 °C for 6 h to consolidate the film.

The SNP laminated membrane on the porous PVDF film substrate (SNP-P) was coated by a procedure described in our previous publications [16,20]. The SNP suspension for coating was prepared by mixing the suspension of activated SNPs in EtOH, PVDF solution in DMSO, and a mixed solvent of DMSO and EtOH. The final suspension contained 0.02 wt.% of SNPs, 0.06 wt.% of PVDF and the balancing EtOH + DMSO mixed solvent. The EtOH and DMSO mixture had an EtOH-to-DMSO weight ratio of 2:1. This SNP suspension composition was determined to ensure complete dissolution of a small amount of PVDF while avoiding high DMSO concentration that would compromise the structure of the PVDF substrate. The suspension was coated onto the surface of a 3.4 cm diameter PVDF sheet (i.e., coating area of 9.1 cm^2^) via complete filtration of 3.4 g (~3.4 mL) SNP suspension assisted by downstream vacuuming. The SNP laminated membrane on PVDF support was then dried in a vacuum oven at 80 °C for 3 h under high vacuum for complete solvent removal. The membrane was further cured at 120 °C for 3 h in the vacuum oven maintained at an absolute pressure of ∼24 kPa.

### 2.5. Membrane Performance for PV of Concentrated Brines

The membrane PV desalination experiments using sweeping gas flow on the permeate side were performed similarly as described in our previous work [16]. Briefly, the disc-shaped membranes were mounted in a Teflon permeation cell using silicon rubber gaskets. The active membrane area was ~2.5 cm^2^ after excluding the sealed edge. The feed solution was circulated at a volumetirc flow rate of 63 cm^3^/min during the tests. Air sweeping rate at the permeate side was ∼250 STP cm^3^/min under ambient pressure. Two different feed solution compositions were used in the membrane PV experiments. The first feed was a 6.77 wt % TDS solution containing 0.20 wt % MgSO_4_, 6.35 wt % NaCl, 0.19 wt % KCl, and 0.03 wt % LiCl; and the second was a 22.04 wt % TDS solution containing 1.98 wt % MgSO_4_, 19.06 wt % NaCl, 0.76 wt % KCl, and 0.24 wt % LiCl. These specific compositions were chosen to reflect produced wastewater from drilling, mining, and CO_2_ geostorage operations, which could be used for lithium extraction if the lithium ions could be further concentrated [21,22]. The membrane PV tests were conducted at a temperature of 73 ± 3 °C. The water vapor at the permeate side was condensed in an ice/water bath (0 °C). Metal ion composition in the condensed liquid was analyzed by inductively coupled plasma-mass spectrometry (ICP-MS, Agilent, Santa Clara, CA, USA 8800 ICP-QQQ). Membrane PV tests were also conducted by pulling vacuum at the permeate side instead of sweeping by an air flow. The membrane PV desalination performance was characterized by the water flux (*J_w_*, kg/m^2^·h) and ion rejection rates (*r_i_*), which are defined by Equations (1) and (2), respectively.

(1) $$J_{w}=\frac{Q_{w}}{A_{m}}\;$$

(2) $$r_{i}=\frac{C_{f}-C_{p}}{C_{f}}$$

where *Q_w_* is the rate of water flowing through the membrane (kg/h), *A_m_* is the active membrane area (m^2^), and *C_f_* and *C_p_* (mol/m^3^) are the metal ion concentrations in the feed solution and product water condensed on the permeate side, respectively.

## 3. Results and Discussion

### 3.1. SNP Preparation

**Figure 1:**
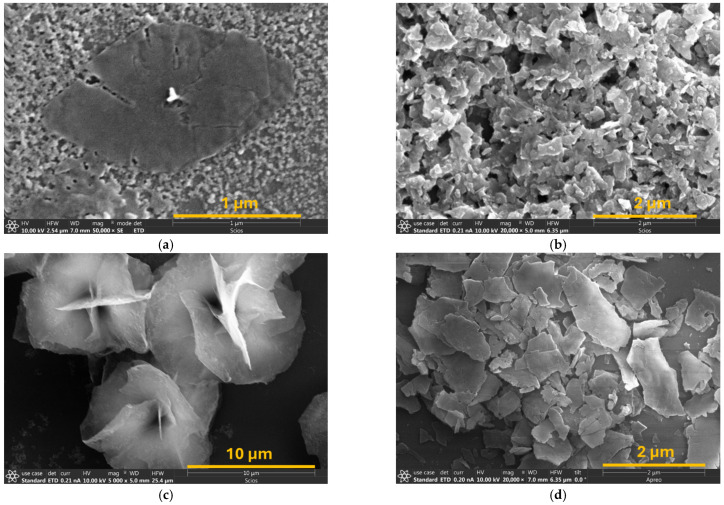
SEM images of (**a**) an as-synthesized SN grown from the spherical seed particle with a thick core in the center, (**b**) flat SNSs separated from the rhombus SNs and ball-milled for seeding the growth of SN assemblies, (**c**) flower-like SN assemblies, and (**d**) SNPs obtained by fragmentation of the flower-like SN assemblies.

Figure 1a shows an SEM image of a single crystalline SN grown from the spherical silicalite nanoparticle seeds in the dC_5_-containing precursor. Each of the typical rhombus nanosheets contains a thick core evolved from the seed, which is undesirable for laminating ultrathin membranes. The flat SNSs obtained by mechanical disassociation, segregation, and ball-milling for seeding the SN assembly synthesis are shown in Figure 1b. Figure 1c shows an SEM image of the flower-like nanosheet assemblies synthesized from the SNS seeds using dC_5_ SDA. After disintegrating the SN assemblies by sonication in the ZrO_2_ milling bead bed, a large amount of flat SNPs was recovered, as shown in Figure 1d. These flat SNPs have been extensively characterized in our previous study [19]. The SNPs separated from the flower-like assemblies had an average thickness of ~60 nm, consisting of multiple layers of 4 nm thick SNS. The SNS thickness is along the *b*-axis orientation, i.e., running in the direction of the 0.56 nm diameter straight channels. The original SNPs from the flower disassociation (Figure 1d) were further processed by sedimentation fractionation to obtain relatively uniform SNPs. The SNPs thus separated for laminating membranes had an average area around 0.5 μm × 1.5 μm. The small SNP fragments separated from large SNPs after disassembling the SN assemblies were directly reused to grow the next generation of SN assemblies in the same dC5-containing precursor. This enables a three-step circular, self-seeded synthesis of SNPs within 7 days [19,20], representing a major simplification compared to the non-circular seven-step SNS synthesis, which requires more than 30 days, consumes additional chemicals, and generates greater chemical waste [13,15]. The flower-like architecture of the SN assemblies prevents irreversible SNP aggregation during calcination, resulting in an overall yield of dispersed open-pore SNPs exceeding 25% [19,20]. In contrast, the thermal activation of SNS produces well-dispersed open-pore SNS suspension with a prohibitively low yield of less than 1% [17].

### 3.2. SNP Laminated Membranes

**Figure 2:**
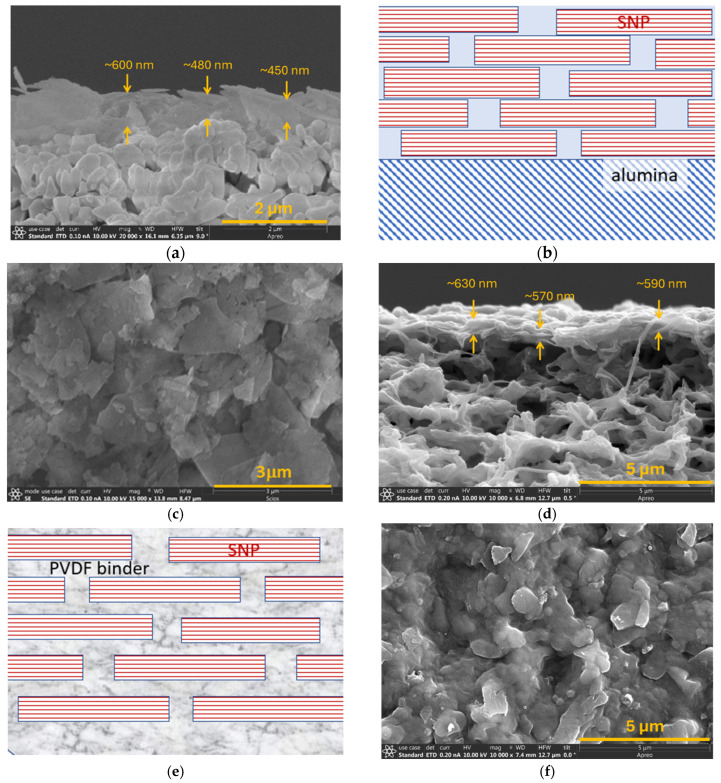
SEM images of (**a**) cross-section of SNP-A, (**b**) schematic depicting the compact SNP layer on alumina after calcination and (**c**) surface of SNP-A, (**d**) cross-section of the SNP-P, (**e**) schematic illustrating increased inter-SNP spaces by the PVDF binder between in the layered SNPs, and (**f**) surface of the SNP-P.

The very low SNP content (0.02 wt.%) in the suspension allowed sufficient sedimentation time for larger-size plates to lie horizontally first on the substrate surface without random packing, which could happen with high-content SNP suspensions. Figure 2a,b are SEM images of the cross-sections of an SNP laminated membrane on alumina substrate (SNP-A) and depiction of the compact multilayered SNPs after consolidation by calcination, respectively. Figure 2c is an SEM image of the SNP-A surface, where very small SNP debris appear on the surface because they are the last to settle after the much larger SNPs had precipitated. Figure 2d,e are a cross-section SEM image of the SNP-P and schematic illustrating the multilayered SNPs bonded by PVDF, respectively. Figure 2f is an SEM image of the SNP-P surface, which exhibits typical texture and morphology of a PVDF-bonded SNP layer on porous PVDF [20]. The SNP laminated membrane thicknesses were about 500 nm on the alumina substrate (SNP-A) and about 600 nm on the PVDF support (SNP-P). Both the SNP-A and SNP-P layers were thicker than the theoretical thickness (~403 nm) of a dense film with equivalent silicalite mass. The obviously thicker SNP layer on the PVDF substrate can be attributed to the PVDF binder between the SNPs which increased the inter-SNP spaces. The HPC binder in the SNP-A was removed during calcination consolidation of the multilayered SNP membrane, resulting in a more compact structure with smaller inter-SNP spaces than the SNP-P. The difference in compactness of the SNP-A and SNP-P was found to be significantly influential on the membranes’ performance in PV for high-TDS solutions.

The SNP-A and SNP-P are also microstructurally different from their counterparts of SNS laminated membranes on the same alumina (SNS-A) and PVDF (SNS-P) substrates, respectively [15,16]. Because the SNS are 4 nm thick single crystal sheets, the multilayered SNS-A, which was consolidated via vapor-phase crystallization and calcination, is more compact with smaller inter-SNS spaces than the SNP-A [15]; and the multilayered SNS-P layer bonded by PVDF binders has significantly more and longer inter-SNS pathways than the SNP-P [16]. The structural differences are also expected to cause performance differences in PV desalination of high-TDS solutions.

### 3.3. PV on SNP Laminated Membranes

**Figure 3:**
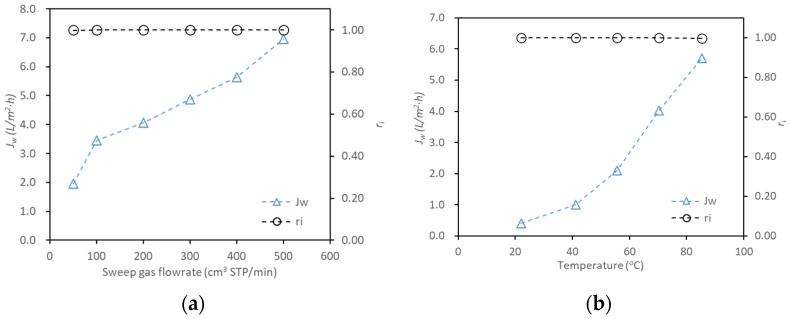
Water flux and salt rejection of the SNP-P during PV desalination of the 6.77 wt.% TDS solution: (**a**) as a function of sweep gas flow rates at a fixed temperature of ~71 °C, and (**b**) as a function of temperatures at a fixed sweep flowrate of ~245 cm^3^ (STP)/min.

An SNP-P membrane with an active area of 2.5 cm^2^ was tested for PV desalination of a solution containing 6.77 wt.% TDS. Experiments were conducted under (i) varied sweep gas flow rates at a fixed temperature of ~70.5 °C, and (ii) varied temperatures at a fixed sweep gas flow rate of ~245 cm^3^ (STP)/min. The results are shown in Figure 3. The membrane was operated for ~18 h at varied sweep flowrates under constant temperature, and for ~44 h at a fixed sweep flowrate with increasing temperature, the latter requiring longer operation at low temperatures to collect sufficient water product for analysis. A largely linear increase in *J_w_* was observed with increasing sweep flowrate, attributed to the enhanced driving force from reduced water vapor partial pressure on the permeate side. *J_w_* increased nonlinearly with rising temperature, resulting from the nonlinear increase in saturation vapor pressure in the feed side and the enhancement of diffusivity across the membrane. Salt rejection remained consistently above 99% throughout the experiments.

The SNP-A and SNP-P membranes were tested at 73 ± 3 °C for PV desalination of the 6.77 wt.% and 22 wt.% TDS solutions using sweeping air and vacuum pressures on the permeate side, respectively. Each PV experiment was conducted for 10–12 h at 73 ± 3 °C, with permeate water samples collected every 2–3 h. Only a moderate decline in flux was observed during the first ~5 h, while ion rejection remained essentially unchanged throughout the process. Therefore, the flux and rejection values reported hereafter represent averages from three to four samples.

**Figure 4:**
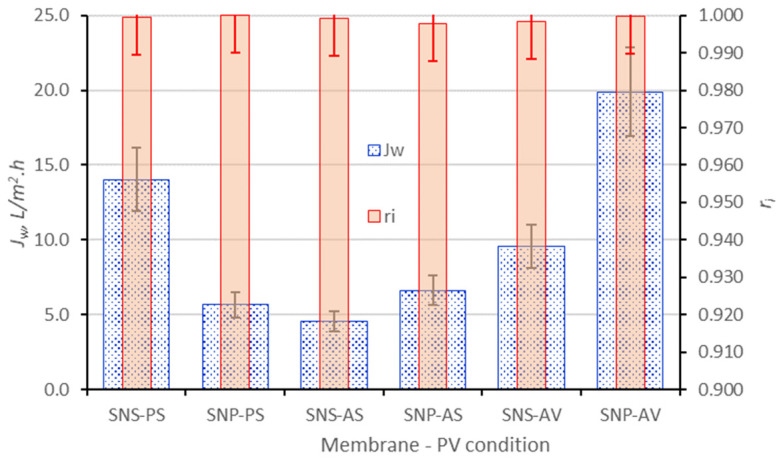
SNP-P and SNP-A membrane PV desalination results for feed solution with 6.77 wt.% TDS in comparison with previously reported results on SNS-A and SNS-P membranes [15]: the last letter “S” denotes operation by sweeping air and “V” denotes operation under vacuum pressure on the permeate side.

**Figure 5:**
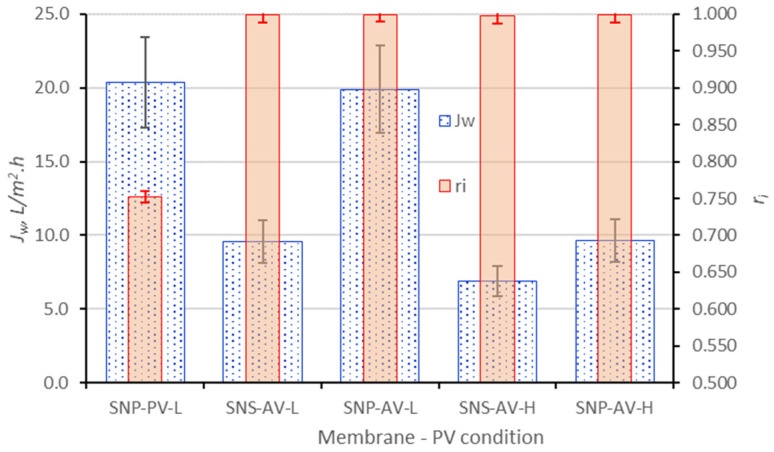
SNP-P and SNP-A membrane PV desalination results for feeds with TDS of 6.77 and 22.04 wt.% TDS under vacuum operation in comparison with the results on SNS-A from a previous report [15]: “V” denotes vacuum, “L” denotes TDS of 6.77 wt.%, and “H” denotes TDS of 22 wt.%.

The PV desalination results, including water flux and total salt rejection, are presented in Figure 4 and Figure 5 together with the data of SNS-A and SNS-P obtained in our previous studies [15,16]. The SNS-A and SNS-P were coated with the same amount of SNS as the SNP membranes that made them comparable here. In Figure 4 and Figure 5, the error bars represent the variations of multiple independent membrane tests, including three SNP-P and two SNP-A samples with multiple tests.

The SNP-A membrane achieved a water flux of 6.6 L/m^2^·h when operating with a sweeping air flow (SNP-AS) and its water flux dramatically increased to 19.9 L/m^2^·h when operating under vacuum pressure on the permeate side (SNP-AV). This may be explained by the fact that, under air sweep, stagnant air filled the small pores (dia. ~0.1 µm) of the thick alumina support that created high resistance to water vapor diffusion. The transport resistance from the stagnant air in substrate was nonexistent under vacuum operation, which led to a drastically higher water flux. In both operations, near-complete salt rejections (>99.8%) were achieved on the SNP-A, indicating that the size-exclusion effects of the zeolite pore openings were effectively achieved by the multilayered SNP membrane. The rejections of individual ions were also similarly near to complete and the rejection of lithium ions by the SNP-A was greater than 99.7% in all cases. The SNP-P membrane, although with a substrate of smaller thickness and larger pore size and porosity than the alumina disc, exhibited slightly lower water flux than the SNP-A, suggesting higher resistance from the thicker SNP layer where the PVDF binder could partially block the zeolitic pore entries. Also, the SNP-P membrane was unable to obtain high levels of salt rejection when operating under vacuum pressure (shown in Figure 5), which was likely due to the solution migration through the inter-SNP pathways spaced by the PVDF binders.

The water flux of the SNP-A was double that of the SNS-A membrane (9.56 L/m^2^·h [15]) under vacuum operation. In the SNP-A, the 60 nm thick SNPs are composed of 4 nm thick SNS layers stacked without blocking the individual SNS surface pore entrances [15]; and the inter-SNP “≡Si-O-Si≡” connections, formed via silanol condensation between SNP surfaces, minimize pore blocking at the microscopically uneven SNP interface. In the SNS-A, the larger number of 4 nm thick SNS layers are consolidated by vapor-phase crystallization of impregnated TPA-templated precursor. These in situ formed crystals strongly bond the SNS stack but orient randomly to partially obstruct the SNS surface pore entrances and thereby reduce the water permeability of the SNS-A membrane. On the other hand, both the SNP-A and SNP-P exhibited water flux lower than the SNS-P membrane (14.03 L/m^2^·h [16]) when operating under sweeping air. These indicate that the isolated SNS allows for faster water transport than the tightly stacked SN layers when pore openings are fully accessible when the binders are more sparsely distributed over the larger inter-SNS spaces. Another factor that helped enhance the water flux of the SNS-P was the small alumina content in the zeolite framework. The alumina-containing nanosheets made the surface more hydrophilic to favor water adsorption into the zeolitic pores. Nevertheless, the water flux of SNP-A under vacuum operation was still 42% higher than the SNS-P under sweeping air.

### 3.4. PV for High TDS Brines

The silicalite membrane’s size-exclusion mechanism for salt rejection and extraordinary hydrothermal stability are particularly desirable for PV of high TDS and at elevated temperatures. The SNP laminated membranes were further evaluated by vacuum PV for the 22.04 wt.% TDS feed. The results of PV at 73 ± 3 °C are presented in Figure 5 together with the results on the SNS laminated membranes reported in a previous publication [15].

The SNP-A membrane achieved near-complete salt rejection for the feed with 22.04 wt.% TDS under vacuum operation. However, the water flux decreased to 9.64 L/m^2^·h from 19.9 L/m^2^·h for the feed with 6.77% TDS. This decrease in water flux was mainly caused by the decrease of PV driving force, i.e., the transmembrane water vapor pressure ${∆P}_{w}=P_{w,p}^{sat}-P_{w,p}$ where $P_{w,p}^{sat}$ and $P_{w,p}$ are the feed side saturation pressure and permeate side vacuum pressure, respectively. The saturation water vapor pressure decreases with increasing TDS which lowers the driving force for PV of high TDS solutions. Also, under high water flux with high TDS feed, the feed side membrane surface and potentially the near-surface inter-SNP spaces could reach oversaturation to cause salt crystallization, preventing liquid migration through the inter-SNP spaces but also leading to partial blockage of zeolite pore entrances [15,16]. The SNP-A outperformed the SNS-A with significantly higher water flux than the latter (6.9 L/m^2^·h) because of the more severe loss of pore entrances by inter-SNS crystallization. It should be noted that the water flux with near-complete salt rejection is among the best of reported membrane desalination performance for similarly high TDS solutions [1,5,8,23]. On the other hand, the SNP-P membrane exhibited an extraordinary water flux (20.4 L/m^2^·h) but with a significantly decreased salt rejection ( $r_{i}$ = 75.2%) even for the solution of 6.77% TDS. This low salt rejection was likely caused by a small amount of solution migrating through the larger inter-SNP spaces. Apparently, such minimal liquid solution permeation was prevented in the SNP-P during sweeping air operation because of overall equal pressures on the feed and permeate sides; however, minimal liquid permeation occurred driven by a transmembrane pressure created by a downstream vacuum pressure.

The comparison of current work with other zeolite membranes for PV desalination is summarized in Table 1. High water flux, e.g., close to 20 L/m^2^⋅h, were observed [5,8]. Those data were collected at much lower feed salt concentrations compared to our tests. There were significant flux drops, sometimes together with increased salt rejection as salt concentration increased [8,23]. The SNP-A membrane demonstrated high flux and great salt rejection even at 22.04 wt.% (260 g/L) of TDS, testifying to its outstanding performance compared to other zeolite membranes.

### 3.5. Membrane Reproducibility

**Figure 6:**
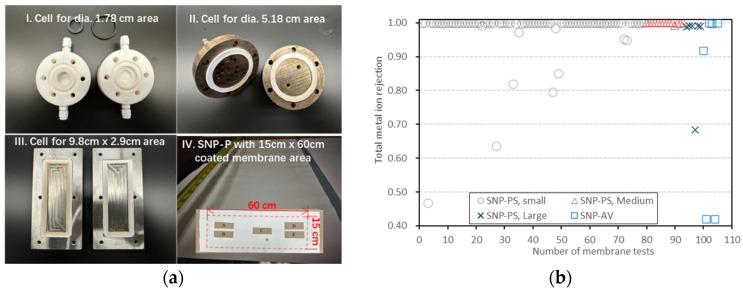
Summary of PV tests of all SNP laminated membranes: (**a**) photos of the PV membrane cells and large-area SNP-P ((**I**)—1.8-cm dia, (**II**)—5.18-cm dia., (**III**)—9.8 cm × 2.9 cm, and (**IV**)—SNP-P with a 15 cm × 60 cm coated area) and (**b**) summary of salt rejection of all SNP laminated membranes tested (“SNP-PS, Small”—1.8-cm dia., “SNP-PS, Medium”—5.18-cm dia., “SNP-PS, Large”—9.8 cm × 2.9 cm samples from the 900 cm^2^ membrane, and SNP-AV—PV rejection for 22.04 wt% TDS under vacuum operation).

In this study, membranes with *r_i_* < 97% were disqualified because salt rejection is critical for ensuring discharge water purity and the retention of valuable materials. Water flux was not considered in this estimate of reproducibility because the sweep gas flowrate was much lower for the larger-sized membrane samples (12 and 28 cm^2^) when normalized by the membrane area due to the apparatus’ capacity limitations. However, the flux variations of the large number of comparable membranes are within ±25%. SNP-P membranes of larger sizes, including active membrane areas of 5.18 cm diameter (21 cm^2^) and 15 cm × 60 cm (900 cm^2^), were fabricated using custom-made flat coaters following the same SNP suspension load per cm^2^ coating area and identical post-coating treatment procedures. The tests of the whole 900 cm^2^ membrane are yet to be performed using modules of properly designed flow fields and increased flowrates of gas and liquid with controlled temperatures. However, the integrity of the larger membranes was demonstrated by preliminary PV tests for samples cut from representative locations of the 900 cm^2^ membrane (Figure 6(aIV)). Figure 6b presents a summary of PV results for all membranes tested in this project, including the 1.8 cm dia., 5.18 cm dia. and 9.8 cm × 2.9 cm (samples from the 900 cm^2^ membrane) SNP-Ps and the SNP-A. Data in Figure 6b also indicate the reproducibility for the SNP-P and SNP-A, which are >90% and ~50%, respectively. It should be noted that the SNP-A data in Figure 6 are for high-TDS (22.04%) PV under vacuum.

The SNP-P membranes demonstrated excellent reproducibility on more than 90% of the tested membrane samples, while the SNP-A membranes had a relatively low success rate of about 50% in reproducing membranes with the performance shown in Figure 4 and Figure 5. The high reproducibility of SNP-P membranes was expected from the self-repairing effect during the filtration–coating process [20]. However, the self-repairing mechanism was less effective in coating the SNP-A membranes, likely due to the incidental entrapment of small, irregular SNP fragments between larger SNPs and the absence of polymer binder filling when such defects formed.

## 4. Conclusions

A new type of multilayered silicalite nanosheet plate (SNP) laminated membrane was fabricated on a porous alumina disc (SNP-A) and PVDF film (SNP-P). The SNP laminated membranes demonstrated excellent capability for PV desalination of concentrated solution containing complex composition with lithium ions. When operating at 73 ± 3 °C with vacuum pressure on the permeate side, the SNP-A membrane achieved nearly perfect salt rejection ( $r_{i}$ ~99.9%) and a remarkable water flux of 19.9 L/m^2^·h, which was significantly higher than that obtained by the SNS-A ( $J_{w}$ = 9.56 L/m^2^·h). On the other hand, when operating by sweeping air flows, the SNP-P membrane exhibited an excellent $J_{w}$ of 14.0 L/m^2^·h and near-complete $r_{i}$ > 99.9% for a feed with 6.77% TDS, outperforming the SNP-A membrane. However, the $r_{i}$ of the PVDF-supported membranes was found to decline when operating under vacuum pressure, likely because of minimal liquid permeation through the inter-SNP spaces driven by the transmembrane pressures. The SNP-A membrane also significantly outperformed the SNS-A membrane with much higher water fluxes in PV operations.

In existing industrial systems, concentration of lithium and rare earth elements from hundreds of ppm to over 1% is typically the first step of production processes. Membrane PV desalination may provide an alternative to the environmentally concerning conventional thermal or solar evaporation concentration methods, with the potential to achieve zero liquid discharge through recycling operations. In this study, SNP-A has demonstrated PV performance suitable for lithium concentration from high-TDS brines with near-total retention. The ceramic-supported SNP membranes, due to their intrinsic resistance to hydrothermal conditions, hypersalinity, dissolved organics, acids, bases, and radiation, can be used for PV desalination to concentrate critical metals from brines and recycled waste streams, especially under conditions unsuitable for polymer membranes. However, ceramic-supported SNP membranes may have affordability issues due to the high cost of substrates, especially in the U.S. The development of more affordable substrates and improvement of SNP-A reproducibility are needed. Furthermore, high-performing vacuum-driven PV operation may involve an increase in energy consumption, which needs to be evaluated and taken into consideration in techno-economic assessment.

## Figures and Tables

**Table 1:** Summary of our membrane’s performance in comparison with other zeolite membranes reported in the literature.

Membranes	Thickness (μm)	Water Flux (L/m^2^·h)	Salt Rejection	Salt Concentration (g/L)	T (°C)	Ref.
MFI (ZSM-5)	0.65	11	99.9%	260	75	[16]
MFI (ZSM-5)	0.41	3.5	99.9%	280	80	[15]
MFI (ZSM-5)	–	0.72	>99%	38	80	[12]
MFI (ZSM-5)	3.3	11.5	96%	3	75	[11]
MFI (Silicalite-1)	–	7	99%	10	60	[24]
MFI (Silicalite-1)	5	13.8	99.8%	10	60	[23]
MFI (Silicalite-1)	5	3.7	94.6%	75	60	[23]
MFI (Silicalite-1)	5	20.6	99.9%	10	70	[8]
MFI (Silicalite-1)	5	12.2	96.9%	70	70	[8]
MFI (Silicalite-1)	7.08	1.22	99.8%	30	80	[25]
LTA (NaA)	3.75	1	99.44%	30	25	[25]
LTA (NaA)	~9	1.9	99.9%	10	69	[26]
FAU	2	4	>99%	35	75	[3]
FAU	0.5	18	99.7%	30	70	[5]
CHA	0.6	13	99.9%	30	70	[5]
AEI (AlPO_4_-18)	15	2.14	99.7%	30	25	[4]
SOD (Sodalite)	1	3.5	>99.99%	10	200	[7]
HEU (Clinoptilolite)	–	2.5	95.8%	0.1	93	[10]
MFI (SNP-A)	0.5	19.9	99.9%	70	73	This work
MFI (SNP-A)	0.5	9.56	99.9%	260	73	This work
MFI (SNP-P)	0.6	14	>99.9%	70	73	This work

## Data Availability

The original contributions presented in the study are included in the article. Further inquiries can be directed to the corresponding author.
