# Correction: Sun et al. Silicalite Nanosheet Laminated Membranes: Effects of Layered Structure on the Performance in Pervaporation Desalination. *Membranes* 2026, *16*, 32

**DOI:** 10.3390/membranes16030103

**Published:** 2026-03-12

**Authors:** Xinhui Sun, Yukta Sharma, Landysh Iskhakova, Zishu Cao, Junhang Dong

**Affiliations:** 1 Department of Chemical and Environmental Engineering, University of Cincinnati, Cincinnati, OH 45221, USA; 2 Jasper Department of Chemical Engineering, The University of Texas at Tyler, Tyler, TX 75799, USA

## Error in Figure

In the original publication [1], there was a mistake in Figure 3b, as published. The wrong figure (duplicate of Figure 3a) was pasted in for Figure 3b during the proofreading process when we were correcting the error of units in the figure. The corrected Figure 3b appears below. 

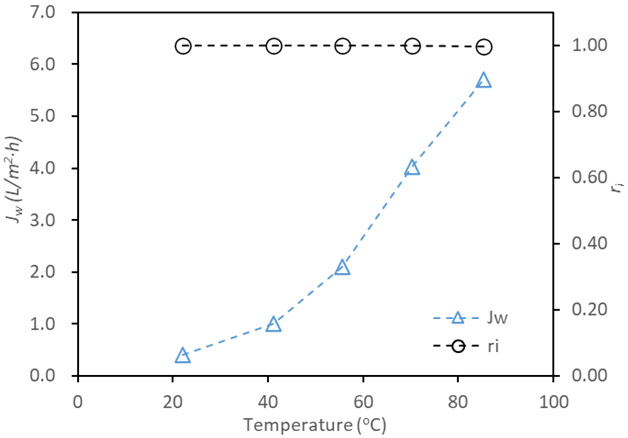



The authors state that the scientific conclusions are unaffected. This correction was approved by the Academic Editor. The original publication has also been updated.
